# Obesity and cancer risk: evidence, mechanisms, and recommendations

**DOI:** 10.1111/j.1749-6632.2012.06750.x

**Published:** 2012-10-10

**Authors:** Ivana Vucenik, Joseph P Stains

**Affiliations:** 1 Department of Medical and Research Technology, University of Maryland School of MedicineBaltimore, Maryland; 2 Department of Pathology, University of Maryland School of MedicineBaltimore, Maryland; 3 Department of Orthopaedics, University of Maryland School of MedicineBaltimore, Maryland

**Keywords:** obesity, cancer, mechanisms, recommendations, prevention

## Abstract

Obesity, a growing health problem worldwide, has been associated with the metabolic syndrome, diabetes, cardiovascular disease, hypertension, and other chronic diseases. Recently, the obesity–cancer link has received much attention. Epidemiological studies have shown that obesity is also associated with increased risk of several cancer types, including colon, breast, endometrium, liver, kidney, esophagus, gastric, pancreatic, gallbladder, and leukemia, and can also lead to poorer treatment and increased cancer-related mortality. Biological mechanisms underlying the relationship between obesity and cancer are not well understood. They include modulation of energy balance and calorie restriction, growth factors, multiple signaling pathways, and inflammatory processes. Key among the signaling pathways linking obesity and cancer is the PI3K/Akt/mTOR cascade, which is a target of many of the obesity-associated factors and regulates cell proliferation and survival. Understanding the molecular and cellular mechanisms of the obesity–cancer connection is important in developing potential therapeutics. The link between obesity and cancer underscores the recommendation to maintain a healthy body weight throughout life as one of the most important ways to protect against cancer.

## Introduction

Obesity and cancer are two major epidemics of this century. Here, we provide an overview of the global obesity and cancer burden, discuss recent advances, and speculate on novel mediators that underpin the molecular mechanisms and challenges in developing prevention and treatment strategies against obesity-related malignancies.

Obesity is one of the most serious public health problems worldwide, even in developing countries. Its prevalence has dramatically increased in the last few decades, reaching epidemic proportions.^1^–^3^ In epidemiological studies, obesity is often defined with body mass index (BMI) as a single measure that can be compared across studies and populations. According to the World Health Organization (WHO) criteria, a BMI greater than or equal to 25 kg/m^2^ is overweight, while obesity is defined as having a BMI equal to or higher than 30 kg/m^2^. The marked increase in the worldwide incidence of obesity, particularly in children, has been noted by the WHO: worldwide there were 1.5 billion overweight and 500 million obese adults in 2008, and nearly 43 million children under the age of five were overweight in 2010. Within the last four decades the prevalence of obese people in the United States increased and is currently 66% of adults with a BMI > 25 kg/m^2^ and half of those have a BMI of >30 kg/m^2^ (Ref. 4). However, according to the latest reports, after 30 years of constant rise, obesity rates in the United States appear to be stabilizing among both adults and children, probably as a result of obesity-prevention initiatives and efforts to address the nation's obesity problem.^4^,^5^

Cancer is the leading cause of death in developed countries and a second leading cause of death in developing countries.^6^,^7^ About 12.7 million cancer cases and 7.6 million cancer deaths are estimated to have occurred in 2008.^7^ The number of cancer cases are expected to increase due to the growing and aging population. In addition, the number of cancer survivors is steadily on the rise. There were approximately 12 million cancer survivors in 2008 as estimated by the National Cancer Institute.^8^

## Health issues related to obesity

Overweight and obesity are major risk factors for noncommunicable diseases, such as cardiovascular diseases (heart disease and stroke), diabetes, osteoarthritis and musculoskeletal disorders, fatty liver, gall stones, psychological disorders, and psychosocial problems.^9^ Increased mortality has also been related to obesity.^10^ Consequently, the obesity–cancer link has recently received much attention.

## Obesity and cancer

Epidemiological studies have shown that obesity is associated with increased risk of several cancer types, including colon, endometrium, postmenopausal breast, kidney, esophagus, pancreas, gallbladder, liver, and hematological malignancy.^1^,^11^ A comprehensive systematic review of the evidence by the World Cancer Research Fund (WCRF) and American Institute for Cancer Research (AICR) concluded that obesity is an established risk factor for several cancers.^1^ In addition, obesity can lead to poorer treatment outcome, worsened prognosis, and increased cancer-related mortality.^12^,^13^

Although the correlation between obesity and cancer can be found in the medical literature going back for several decades, the relationship between obesity and cancer was poorly understood until Calle *et al.*^14^ conducted a large prospective study examining the role of obesity or excess adiposity in increasing the risk of dying from most types of cancer. Increased body weight was associated with increased death rates for all cancers combined and for cancers at multiple specific sites. Obesity has increased the death rate in the United States by 52% in men and 62% in women.^14^ Further attempts have been to made (e.g., Calle and Kaaks^15^) to define why obesity is such an important determinant of cancer risk.

## Potential mechanisms linking obesity to cancer

Biological mechanisms underlying the relationship between obesity and cancer are complex and not well understood. They include obesity-related hormones, growth factors, modulation of energy balance and calorie restriction, multiple signaling pathways, and inflammatory processes,^15^–^21^ affecting cancer cell promotion and progression.

It has been established that fat tissue is an endocrine organ that produces and secretes polypeptide hormones, adipokines, among which leptin and adiponectin are most abundant and involved in cancer development.^22^ Leptin is positively correlated with adipose stores and nutritional status, and important in energy balance and apetite control. Leptin has been extensively studied as a potential mediator of obesity-related cancer.^16^ It induces cancer progression by activation of PI3K, MAPK, and STAT3 pathways.^20^,^23^,^24^ Adiponectin is secreted mostly from visceral adipose tissue and, contrary to leptin, is inversely associated with adiposity, hyperinsulinemia, and inflammation.^17^ Moreover, adiponectin may exert anticancer effects by decreasing insulin/insulin-like growth factor (IGF)-1 and mTOR signaling via activation of 5′AMP-activated protein kinase (AMPK) and exerting anti-inflamatory actions via the inhibition of nuclear factor kappa-light-chain-enhancer of activated B cells (NF-κB).^17^

Body adiposity is associated with higher levels of proinflammatory cytokines, including prostaglandin E2, TNF-α, IL-2, IL-8, IL-10, and monocyte chemoattractant protein (MCP)-1. Activation of NF-κB complex is a possible mechanism through which inflammation may stimulate cancer development.^19^,^21^

Steroid hormones, including estrogen, progesterone, androgens, and adrenal steroids are associated with energy balance and obesity-related progression of several types of male and female cancer.^25^ Adipose tissue can produce estrogens via aromatase-catalyzed conversion of gonadal and adrenal androgens to estrogen in men and postmenopausal women.^26^

Notably, insulin and IGF are now at the center of a growing wave of research around the world aimed at elucidating what many scientists consider to be critical factors in fueling a wide range of cancers.^27^ And now that obesity, diabetes, and cancer rates have each increased substantially, the need to understand this link has become far more urgent.

Human observational studies have reported increased cancer mortality in those with obesity and type 2 diabetes, which may be attributable to hyperinsulinemia, elevated IGF-1, or potentially both factors. Conversely, those with low insulin, IGF-1, and IGF-2 levels appear to be relatively protected from cancer development.^18^ It has been shown that type 2 diabetic patients who get insulin therapy or drugs to stimulate insulin secretion have a significantly higher incidence of cancer than those who get metformin, the antidiabetic drug that works to lower insulin levels.^28^ Epidemiological studies have consistently associated metformin use with decreased cancer incidence and cancer-related mortality, and metformin is rapidly emerging as a potential anticancer agent.^28^ Indeed, these effects underscore the importance of insulin, IGF-1, and energy metabolism in cancer biology. In contrast to the effects of obesity, caloric restriction, which reduces circulating insulin and IGF-1, is a potent suppressor of the carcinogenic process.^29^ Although in animal models of caloric restriction, restoration of circulating IGF-1 is sufficient to abrogate the anticancer action of reduced caloric intake.^30^–^32^

**Figure 1:**
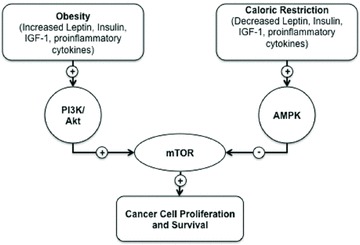
Energy metabolism. Obesity increases circulating leptin, available IGF-1, and proinflammatory cytokines, leading to increased signaling through the PI3K/Akt cascade. These signals converge on mTOR, promoting cell proliferation and inhibiting apoptosis. Conversely, caloric restriction enhances signaling through AMPK, suppressing mTOR activity and promoting cancer cell death.

The role of insulin and IGF as cancer accelerants is a relatively new idea. Insulin and IGF-1 signal through the Akt/PI3K/mTOR cascade to promote cell growth and proliferation, while inhibiting cell survival.^33^ Notably, the Akt/PI3K/mTOR cascade is also one of the signal mediators of obesity-associated factors, such as leptin, adiponectin, and inflammatory cytokines^20^ and is the most frequently mutated pathway in human cancers.^20^,^34^,^35^ Accordingly, while certainly not the only pathway of interest, the Akt/PI3K/mTOR cascade has become a focus of the obesity and cancer connection. This pathway is activated by both insulin and IGF-1, which are frequently present at high levels in the serum of the overweight and obese, leading to enhanced PI3K/Akt/mTOR activation (Fig. 1).^36^–^38^ Conversely, caloric restriction, which is frequently associated with a decreased cancer incidence, reduces PI3K/Akt/mTOR pathway activation at least in part via AMPK activation,^36^,^39^ while tumors with PI3K activation are resistant to the anticancer action of caloric restriction.^35^ Indeed, disruption of the Akt/PI3K/mTOR cascade has been effective at disrupting carcinogenesis and tumor incidence in humans and in animal models.^40^–^43^ However, more agents are needed that can effectively target the molecular mediators of the effects of obesity on cancer progression. We speculate that an understudied, potentially important signaling pathway influencing this cascade may be the inositol polyphosphates.

Inositol is a glucose isomer that can be modified by phosphorylation to serve as a second messenger in cells. The most widely studied of the inositol phosphates is inositol 1,4,5 triphosphate, or IP_3_, which signals the release of calcium from intracellular stores. However, additional inositol polyphosphates exist and have biologic function inside of cells. Indeed, the inositol phosphate multikinase (IPMK), which generates IP_4_ and IP_5_ and functions as a PI3K, has been implicated in growth and metabolism.^44^ Interestingly, in mice fed a high-fat diet, genetic ablation of the inositol hexakisphosphate kinase 1 (IP_6_K1), which produces the inositol pyrophosphate IP_7_, leads to insulin-sensitivity (normal blood glucose, despite decreased circulating insulin), and resistance to obesity.^45^ This effect is mediated by the action of IP_7_ as an inhibitor of the Akt and mTOR signaling downstream of insulin. It would be of great interest to see if IP_7_ signaling downstream of IP_6_K1 could uncouple the link between obesity, regulation of the Akt cascade, and tumorigenesis.

Another kinase responsible for the production of IP_7_, IP_6_K2 has also been implicated in tumor biology.^46^,^47^ IP_6_K2 is a regulator of apoptosis, affecting signaling through Akt and p53.^48^–^50^ Deletion of IP_6_K2 predisposes to certain carcinomas^51^ and has been shown to sensitize tumors to chemotherapeutic treatments.^52^,^53^ Future studies are needed to determine if this interesting group of molecules can be modulated to affect the coupling of obesity and the carcinogenic process.

## Challenges in prevention and treatment

### Guidelines and recommendations for obesity and cancer prevention

It has been estimated that half of the cancers occurring today are preventable by applying knowledge that we already have.^54^ About one-third of cancers in high-income Western countries are attributable to factors relating to food, nutrition, and physical activity.^1^,^54^,^55^ Although diet, obesity, and physical inactivity are modifiable causes of cancer, it is challenging to identify with confidence specific associations between these factors and cancer over a lifetime, because of the long latent period for cancer development and its complex pathogenesis.

The World Cancer Research Fund/American Institute for Cancer Research (WCRF/AICR) Second Expert Report is a major comprehensive, evidence-based document on the relationship between diet, physical activity, and cancer prevention, with recommendations for public health and personal goals.^1^ To address this complex association between food, nutrition, physical activity, body fat, and cancer, the report used a newly developed precise and reproducible method, with a protocol for standardizing the literature search and for analysis and display of the evidence.^1^,^56^,^57^ Nine independent academic institutions worldwide and 17 cancer centers conducted systematic reviews on the causal relationship between food, nutrition, and of weight gain and obesity. A panel of 21 international experts in nutrition, cancer, and obesity, reviewed the evidence, drew conclusions, and made recommendations.^1^,^56^,^57^ On the basis of evidence graded as “convincing” or “probable,” a series of 10 recommendations to reduce the risk of developing cancer was made. One of the most important factors is maintaining a healthy weight throughout life, which can be achieved by regular physical activity and limiting the consumption of energy-dense foods and sugary drinks. Other important dietary measures include consuming a diet high in plant-based foods, limiting intake of red meat, and avoiding salty foods and processed meat. Alcohol should be consumed in modest amounts, if at all. Dietary supplements are not recommended for cancer prevention. Special recommendations were produced for breastfeeding and cancer survivors. For mothers, recommendations were made to breastfeed exclusively up to six months, because lactation protects mothers against breast and possibly ovarian cancer and protects children against overweight and obesity. After treatment, cancer survivors should follow the recommendations for cancer prevention.^1^,^56^,^57^ Although the report was published in November 2007, with the continuous update process (CUP) (http://www.dietandcancerreport.org/cup/index.php), which is now underway and used to keep evidence current, the findings and recommendations remain valid today.^1^,^56^,^57^

In 2009, an evidence-based Policy Report was published, as a companion to the Second Expert Report (http://preventcancer.aicr.org/site/PageServer?pagename=research-science-policy-report). The Policy Report sets out recommendations to all sectors of society to work together. Also, the report estimates the cancer preventability (PAFs) by appropriate food, nutrition, physical activity, and body fat in four countries as representatives of high (the United States and UK), middle (Brazil), and low-income countries (China).

The WCRF/AICR recommendations (http://www.dietandcancerreport.org) are very similar to recommendations coming from the American Cancer Society (ACS) (http://www.cancer.org). The National Expert Panel reviewed evidence and gave recommendations for individual choices and community actions. Namely, the panel suggests that it is important to maintain a healthy weight throughout life and adapt to a physically active lifestyle, to consume a healthy diet based on plants, and limit the consumption of alcohol. For community action, public, private, and community organizations should work together to increase access to healthy foods and provide an environment for physical activity.

Therefore, by alterations in individual and population behaviors, and by public health efforts and social commitment, we can achieve prevention of cancer and chronic disease. However, the major challenge and obstacle to apply what we know about cancer and obesity prevention to society is accountability and to identify who is responsible for addressing these public health issues.^54^

### Challenges in treatment of obese cancer patients

Although obesity is a well-established risk factor for several cancers, its role on cancer survival and recurrence is poorly understood.^13^ The population of cancer survivors, defined as individuals with a cancer diagnosis regardless of the course of the illness until the end of life, is steadily on rise. Epidemiological evidence shows not only that people who are obese and overweight are at increased risk of developing cancer, but also that obesity can affect tumor progression, and it is challenging to define potential ways to intervene to improve outcomes for patients with cancer.^12^,^13^

There are several challenges in the various treatment modalities of cancer in obese patients. Oncologic considerations are focused on chemotherapy and radiotherapy in the obese cancer patients.^12^ Doses of chemotherapeutic agents are usually calculated according to the patient's actual body weight, and there are concerns of relative overdosing of chemotherapy in the obese cancer patients.^12^,^58^ When adjusting the cytotoxic drug dose to avoid toxicity and suboptimal dosing, numerous factors must be taken into consideration, including bioavailability and clearance of the chemotherapeutic drugs.^12^ The radiotherapy of obese cancer patients can be challenging, with technical difficulties in positioning patients in the accelerators and adjusting the treatment volume.^12^,^59^

In addition, the development of effective drugs targeting the molecular mediators of the link between obesity and cancer may have a profound impact on the incidence and treatment of cancer. Further, understanding these molecular players will likely lead to innovative strategies to effectively target the unique energy requirements of cancer cells, permitting effective anticancer treatment while mitigating damage to normal cell function.

The rising incidence of obesity presents a challenge for surgical treatment and considerations.^12^ Several studies have investigated the association between BMI and operative outcome, showing mixed results, from no difference in operative outcomes to high BMI being strongly predictive of worse operative outcomes.^12^,^60^,^61^

## Conclusion

Obesity is associated with increased risk of numerous cancers by various mechanisms. A better understanding of mechanisms by which obesity may influence cancer development and progression is important to develop strategies for prevention of obesity and cancer and to improve outcomes for obese cancer patients. A high proportion of obesity and cancer globally may be prevented by individual and population behavior changes, for which we need a coordinated action of policymakers, educators, and health professionals.
